# Supplementary material for: Mechanisms governing the pioneering and redistribution capabilities of the non-classical pioneer PU.1
Source: Nat Commun. 2020 Jan 21;11:402. doi: 10.1038/s41467-019-13960-2 (PMC6972792; doi:10.1038/s41467-019-13960-2)
Supplement: Supplementary file 7 — Source data [file 41467_2019_13960_MOESM7_ESM.zip › Source_Data/Figure5/Figure5A_MotifScanOutput/homerResults/motif13.similar.html]

motif13

## Information for motif13

A
C
T
G
A
C
T
G
A
G
T
C
C
G
A
T
A
C
T
G
A
G
T
C
A
C
G
T
A
C
G
T
A
C
G
T
A
C
T
G
A
C
G
T
A
C
G
T
A
C
G
T
C
G
T
A
  
Reverse Opposite:  

A
C
G
T
C
G
T
A
C
G
T
A
C
G
T
A
A
G
T
C
C
G
T
A
C
G
T
A
C
G
T
A
A
C
T
G
A
G
T
C
C
G
T
A
A
C
T
G
A
G
T
C
A
G
T
C
  

|  |  |
| --- | --- |
| p-value: | 1e-54 |
| log p-value: | -1.247e+02 |
| Information Content per bp: | 1.975 |
| Number of Target Sequences with motif | 89.0 |
| Percentage of Target Sequences with motif | 2.95% |
| Number of Background Sequences with motif | 104.8 |
| Percentage of Background Sequences with motif | 0.23% |
| Average Position of motif in Targets | 171.0 +/- 96.6bp |
| Average Position of motif in Background | 224.1 +/- 136.6bp |
| Strand Bias (log2 ratio + to - strand density) | 0.6 |
| Multiplicity (# of sites on avg that occur together) | 1.00 |
| Motif File: | file (matrix) reverse opposite |

### Similar de novo motifs found

|  |  |  |  |  |  |  |  |
| --- | --- | --- | --- | --- | --- | --- | --- |
| Rank | Match Score | Redundant Motif | P-value | log P-value | % of Targets | % of Background | Motif file |
| 1 | 0.962 | A T C G A G T C G C A T A C T G A G T C A C G T A C G T A C G T C A T G A G C T A G C T A C G T C G T A | 1e-52 | -121.936337 | 2.72% | 0.19% | motif file (matrix) |
| 2 | 0.813 | A T C G A G T C A C G T C G A T A C G T A C T G A C G T A C G T A C G T C G T A A G T C | 1e-46 | -107.271597 | 2.62% | 0.22% | motif file (matrix) |
| 3 | 0.781 | A C T G A C G T T C G A T G C A C T G A T G A C G C T A T G C A C T G A T A C G G A T C A T C G | 1e-46 | -106.751036 | 2.62% | 0.22% | motif file (matrix) |
